# Factors affecting the quality of life after ischemic stroke in young adults: a scoping review

**DOI:** 10.1186/s12955-023-02090-5

**Published:** 2023-01-19

**Authors:** Elena Gurková, Lenka Štureková, Petra Mandysová, Daniel Šaňák

**Affiliations:** 1 Department of Nursing, Faculty of Health Sciences, Palacký University Olomouc, Hněvotínská 976/3, 775 15 Olomouc, Czech Republic; 2 Comprehensive Stroke Center, Department of Neurology, Palacký University Medical School and Hospital, Olomouc, Czech Republic

**Keywords:** Young ischemic stroke, Quality of life, Health-related quality of life, Functional outcome, Post-stroke depression, Fatigue

## Abstract

**Purpose:**

To synthesize the body of knowledge on the factors influencing the quality of life (QoL) after ischemic stroke (IS) in young adults.

**Methods:**

Guidelines regarding the scoping review methodology developed by the Joanna Briggs Institute, and the PRISMA-ScR checklist for a scoping review was used in this paper. A total of 1197 studies were identified through a bibliographic search in Web of Science, MEDLINE, PsycInfo, ScienceDirect, Scopus, and ProQuest Science Database. Articles published between the years 2000–2021 were included.

**Results:**

A total of nine papers were finally selected to respond to the research question. Three studies were prospective longitudinal studies compared QoL between young stroke and age-matched controls from the general population. Across all the analysed studies, 14 variables potentially associated with QoL were identified. QoL in young patients is mainly affected by clinical outcomes after IS (scored by the modified Rankin scale and the Barthel index—favourable initial functional status and higher independence in ADL leads to higher QoL) and psychological factors (post-stroke fatigue and depression—higher levels of fatigue and depression lead to lower QoL). The reviewed studies emphasized the importance of functional outcomes, post-stroke depression, fatigue and anxiety and early return to work.

**Conclusion:**

Further longitudinal studies are needed to identify the trajectory of post-stroke psychosocial symptoms over time and other potential predictors of unfavourable long-term QoL, thus specific young stroke rehabilitation and stroke self-management support programmes should be developed (address physical, psychological factors which influence the psychosocial adaptation post-stroke and the perception of the QoL).

**Supplementary Information:**

The online version contains supplementary material available at 10.1186/s12955-023-02090-5.

## Introduction

Ischaemic stroke (IS) is traditionally considered a disease of middle-aged and elderly patients. In the literature focused on IS in the young population, there is no uniform age cut-off to define “young adults” [1–5]. Lower age limits range from 15 and 18 years [2]. The most pronounced inconsistencies relate to the upper age limit for IS in young adults [2, 3, 5]. The recent reviews used a limit of 45 years [5] or 50 years [1, 3]. On the other hand, some previous studies used the upper age limit of 55 years [3] and the World Health Organisation (WHO) used even a cut-off of 65 years for the upper age limit in the Global Burden of Disease analyses [6–8]. Epidemiological evidence published in the recent narrative reviews highlighted the increasing incidence of IS in young adults and subsequently the increasing number of young patients who live with physical and psychosocial sequels after IS [1–5].

The incidence of IS in young patients has a global increasing trend with higher mortality and morbidity and risk of recurrence [1]. The prevalence of IS in young adults in the US accounts for approximately 10–15% of all strokes [2, 5, 9]. Incidence in Europe ranges between 3.4–21.7/100,000 and the lowest one is in Northern Europe (10.8–11.4/100,000) [4, 10]. Higher IS incidence in young patients was reported previously in the Hispanic population (26/100,000) [11] and in African Americans in the USA (96/100,000) [12]. A sex difference in IS incidence in young patients was also observed [13].

Stroke outcomes in young patients are generally favourable with a high rate of a good 3-month functional outcome and with lower short-term mortality compared with older stroke patients [2, 14, 15]. Nevertheless, young IS patients may face psychological tasks and concerns about their future and the risk of IS recurrence during the period of “active” life (society, family, and work) [7, 14, 15]. Several quantitative studies with an observational design focused on the long-term issues relating to young stroke emphasized a complexity of problems or “invisible dysfunctions” perceived by young individuals, including fatigue [16], cognitive impairment [17–19], fear from stroke recurrence [20], anxiety, depression [21–23], sexual dysfunctions [24], loss of employment [25–28], family conflicts, social isolation, lack of specialist support, reduction in mobility and life roles, negative body image, and impaired self-efficacy and self-esteem [25, 26].

IS at a young age may have a long-term impact on a patient’s multidimensional health-related quality of life (HRQoL), with an accompanying socioeconomic burden [2–8, 14, 29]. However, there is a paucity of comprehensive information regarding post-stroke HRQoL trajectories in young adult stroke survivors and therefore much of the variance of HRQoL after IS remains unexplained in this population. Existing literature reviews [1–5] and prospective cohort studies [30–34] provide information predominantly about the aetiology, risk factors and prognosis of IS in young adults. Moreover, most of the current understanding of the long-term consequences of IS comes from older populations [5]. A considerable number of systematic reviews [35–38] focused mainly on post-stroke trajectories and predictors of the HRQoL in the older population. In addition, the previous studies and their syntheses evaluating HRQoL after a stroke at a young age were heterogeneous regarding the type of stroke included or the age limits definitions for young adults [39–41]. The only available review [41] describing the determinants of the HRQoL did not specifically address the issue of HRQoL after IS but it comprised studies with other subtypes of juvenile stroke and focused on HRQoL and resilience. This review also did not provide evidence to address the impact of physical consequences of IS on the HRQoL in this specific age group.

HRQoL has been widely recognised as one of the key indicators to measure post-stroke outcomes [42]. Further investigation of predictors of post-stroke HRQoL in young-onset patients is needed for the creation, implementation and evaluation of specific young stroke rehabilitation and stroke self-management programs. Furthermore, the evaluation of currently used HRQoL measures should be performed to identify those measures that accurately reflect the complexity of problems in this specific age group and that could be used in stroke self-management and rehabilitation of young adults with IS [43].

In this review, we aimed to focus comprehensively on differences between younger and older individuals regarding HRQoL and the main factors contributing to HRQoL in young adults with IS.

## Methods

Guidelines for the scoping review (ScR) methodology developed by the Joanna Briggs Institute (JBI) [44], and the PRISMA-ScR checklist [45, 46] for a scoping review were used.

### Review questions

The review addressed the primary (overarching) question: What is known about the HRQoL in young adult stroke survivors?

The specific research questions that guided the scoping review were:

- What are the differences in the HRQoL between younger and older stroke survivors and between young adult stroke survivors and age-matched controls?
- How has the HRQoL been assessed, and which instruments have been used to measure HRQoL in young adult stroke survivors?
- What are the predictors of quality of life in young adult stroke survivors?

A bibliographic search of the literature was conducted from May to June 2021. The relevant studies were identified based on a search of publications between 2000 and May 2021. The ‘‘PCC’’ mnemonic (population, concept, and context [44]) was used to identify meaningful criteria for this scoping review.

### Inclusion criteria

*(a) Participants:* This review has mentioned include the adult population only, and the age range between 18 and 65 years was applied for “young stroke” age definition in this review. Studies with individuals over 65 years at the time of the index IS were excluded, but relevant studies comparing the young and the old stroke patients were included. Studies involving participants with a clinical diagnosis of transient ischemic attack (TIA) or other stroke types than IS were excluded. However, relevant heterogeneous studies comprising a sample of individuals with IS as well as other stroke types were considered if a substantial part of the sample (more than 80%) consisted of young adults with IS.

*(b) Concepts:* HRQoL was the central concept investigated in this scoping review [37]. Thus, we included studies focused on the health aspect of QoL, long-term functional disabilities and physical and psychosocial consequences of IS to cover comprehensively multidimensionality or all relevant domains of HRQoL in young stroke. Studies using stroke specific or generic HRQoL measures were considered. Therefore, studies focused on functional or health status only and did not explore QoL as the main outcome were excluded.

*(c) Context:* The studies were included if conducted in long-term period after stroke or in chronic phase of stroke.

*(d) Type of studies:* After the search was done, quantitative studies with an observational design were included in the analysis. Studies focused on HRQoL measured by standardised questionnaires only were considered. Therefore, qualitative studies did not clearly respond to the inclusion criteria and were excluded from this review. Qualitative studies have explored particular lived experiences of stroke from the perspective of young adults (e.g., the coping experiences [47], the perceived needs and priorities [48], the experience of parenting [49], the lived experiences during the transition period from hospital discharge through the first weeks at home [50], the experiences of QoL in the recovery process across countries [51], the roles of service provision and return to work [52] etc.). Published protocols of studies, discussion papers, reviews, editorials, conference abstracts, books, reports, and dissertations were excluded. Grey literature was also excluded.

### Search strategy

The following search terms and their combinations were identified to cover all long-term consequences of IS according to previously published reviews [1–5]:

*Participant:* (‘stroke’ OR ‘cerebrovascular stroke’ OR ‘cerebrovascular accident’ OR ‘ischemic stroke’ OR ‘ischaemic stroke’) AND (‘young’ OR ‘adult, young’ OR ‘young adult’ OR ‘middle-aged adults’ OR ‘working age’ OR ‘stroke patient’ OR ‘post-stroke’ OR ‘stroke survivor’).

*Concept:* (‘quality of life’ OR ‘health-related quality of life’) AND (‘health status’) AND (‘functional status’ OR ‘activities of daily living’ OR ‘functional disability’).

*Context*: ‘long-term’ OR ‘stroke care’ OR ‘post stroke care’ OR ‘prognosis’

The electronic databases Web of Science, MEDLINE (Ovid), PsycInfo (EBSCO), ScienceDirect (Elsevier), Scopus (Elsevier), and ProQuest Science Database were used to gather data for a review of quantitative studies. Selection terms were modified for use in each database (Additional file 1).

### Study selection

**Fig. 1:**
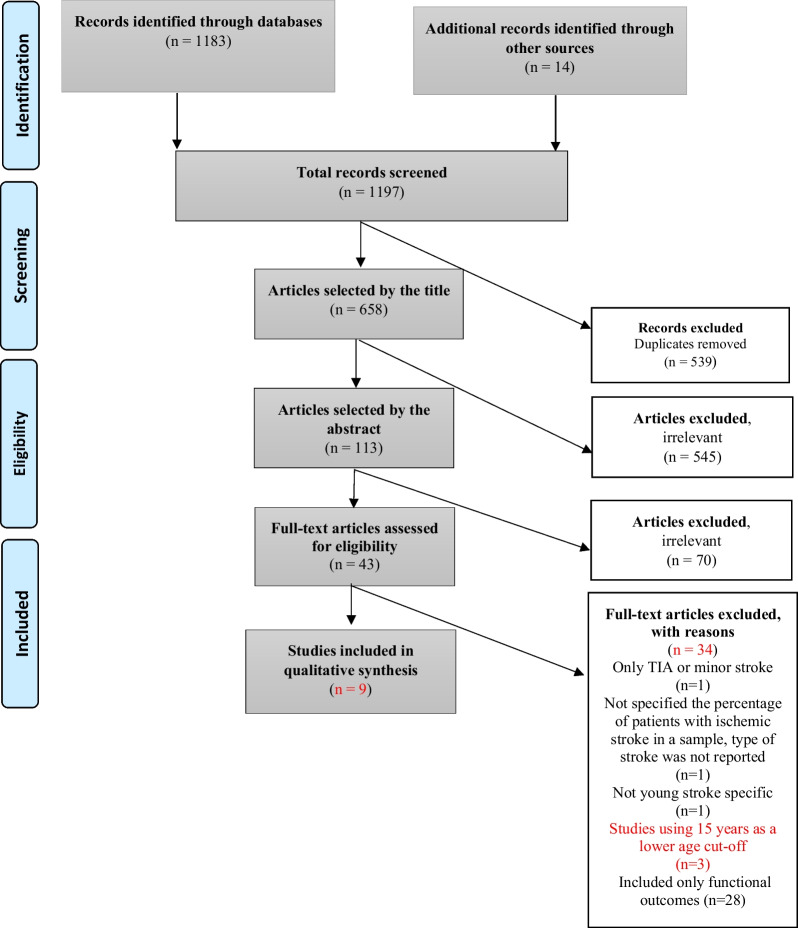
Flow diagram of the study selection process

All citations of the identified records were uploaded into a web-based reference manager, and duplicates were eliminated. Two researchers (E.G., L.Š.) screened studies using the titles, and selected studies were analysed using the abstracts based on the pre-specified inclusion/exclusion criteria. Eligibility and data extraction were conducted by two independent researchers (E.G., L.Š.). The full texts of selected sources were then studied by two researchers (E.G., L.Š.) for the final inclusion in the scoping review. A third researcher (D.Š.) evaluated the studies without the previous agreement about the inclusion (Fig. 1). A disagreement about individual study inclusion was solved by the joint discussion until a consensus was reached.

### Data extraction and analysis

The extracted data included the following information: the author, year, country of origin, study aim, study design, participants and sampling, age mean, assessment tool, measure time (follow-up) and main findings. The data extraction was performed by three researchers; two researchers extracted data (E.G., L.S.) and one researcher (P.M.) cross-checked the extraction. Data were subsequently analysed and reported thematically with the stated research questions [53].

## Results

In total, the search produced 1197 sources, 658 studies were screened using the titles, and a total of 113 studies were analysed using the abstracts based on the pre-specified inclusion/exclusion criteria. The full texts of 43 sources were studied and 9 studies were finally included in the scoping review (Fig. 1).

### Characteristics of included studies

Studies included in this review were conducted across multiple countries and most were carried out in European countries. Four studies included patients only with IS [55, 57, 59, 60]. Five studies included patients with IS and haemorrhagic stroke [20, 26, 54, 56, 58], and the proportion of patients with IS in such studies ranges from 77.7% [20] to 92.4% [26]. Eight studies recruited participants from stroke hospital registries, and one study recruited patients from a national stroke clinical registry [54]. The number of participants in the follow-up (FUP) assessment ranged from 63 [56] to 5154 [54]. Three studies assessed the HRQoL within the time of FUP ≤ 12 months [54, 55, 60]. The other studies evaluated the HRQoL within the time of FUP between one to 6 years (Tables 1, 2, 4).

**Table 1:** Differences in QoL between young stroke patients and age-matched controls from the general population

Author/Year/Country	Study design	Participants and sampling	Age mean (SD), range or median (years)	HRQoL measure	Measure time (Time of assessment after stroke)	Main findings and conclusion
de Bruijn et al. (2015) [57] The Netherlands	Prospective study	Young ISs (18–49 years): 170 Controls: 61	*Age at a stroke*: 41.4 (7) *Age at follow-up*: 46.3 (7.1) Controls: 48 (6.6)	WHOQOL-BREF 26	A mean time to FUP: 4.9 years	There were no statistical differences in QoL between young adults with IS and controls after long-term follow-up Fatigue, depression, anxiety, and unemployment affect the QoL in young adults with IS of mild severity The stroke-specific factors were not confirmed as significant contributing factors of QoL in young adults with IS
Palmcrantz et al. (2014) [58] Sweden	Prospective study	Young adult (< 65 years):150 (80% ISs) Controls: 2661	*Young adult* Mean: 57 (6) Median: 59 IQR: 54–62 *Controls* Median: 46, IQR: 38–55	MYS questionnaire (body functions, limitations, restrictions, personal and environmental factors) EQ-5D	< 6 years after stroke	The negative effects of stroke, on self-rated global health among young individuals living in the community, appear to be substantial, multifactorial and long-standing. The young individuals with IS reported significantly lower health status regarding mobility, self-care, usual activities, anxiety, and depression than the matched general population. Limitations and restrictions regarding previous leisure activities and returning to work were the main predictors of the HRQoL in young individuals with IS
Schneider et al. (2021) [59] Estonia	Prospective study	Young ISs (18–54 years): 352 Controls (18-64 years): 2304	*Age at stroke* Median: 48.8 Range: 19.2–54.9 *Age at follow-up* Median: 54 Range: 27–65 *Controls*: Median: 47 Range: 27–64	EQ-5D-3L	A mean time to FUP: 5.7 years	ISs reported significantly lower health status regarding mobility, self-care, usual activities, anxiety, and depression than controls. The most significant differences between young stroke patients and their non-stroke counterparts were in the physical domain. Lower QoL of young stroke patients was predicted by stroke-specific (coronary heart disease during the index event, longer follow-up duration, recurrent stroke, functional disability) and psychosocial factors (depression, unemployment). Young patients with excellent recovery after IS reported higher QoL than the controls. Young ISs have long-term decreased HRQOL, except for those with excellent functional recovery

*EQ-5D-3L* EuroQoL-5 dimension-3 level, *EQ-5D* EuroQoL-5 dimension, *FUP* follow-up, *ISs* Ischemic stroke survivors, *IQR* Inter quartile range, *mRS* Modified Rankin Scale, *MYS* Mapping Young persons with Stroke, *WHOQOL-BREF 26* shortened World Health Organization Quality of Life scale

**Table 2:** HRQoL and related outcomes

Author/Year/Country	Aim	Study design	Participants and sampling	Age mean (SD), range or median	HRQoL measure	Measure time (Time of assessment post-stroke)	Main findings and conclusion
Rhudy et al. (2020) [60] USA	To investigate QoL in young adult stroke survivors at baseline and 6 months post-discharge	Prospective study	ISs (18–65 years):18	55.56 (9.45) Range: 34–64	PROMIS and NeuroQoL	6-month	The statistically significant improvement from baseline to 6-month follow-up was found only in independence in ADL and cognitive function
Westerlind et al. (2017) [26] Sweden	To explore factors affecting the return to work after stroke	Prospective study	Young adult (18–63 years): 211 (77.7% ISs)	Median: 53 ≤ 50 years: 41.2% ≥ 51 years: 58.8%	*FUP assessment* EQ5D	A mean time to FUP: 6 years	Young ISs who did return to work reported higher VAS scores. There were no significant differences regarding the index value calculated from EQ-5D
Yoon et al. (2021) [20] South Korea	To test a predictive model of QoL in young adults with stroke	Cross-sectional study	Young adult (18–49 years): 237 (92.4% ISs)	46.7 (4.70) Range: 20–49	SS-QoL	A mean time since the stroke: 19.3 (± 13.1) months	QoL was mainly influenced by stroke severity, social support, depression, functional disability, fear of stroke recurrence and perception of health status

*ADL* activities of daily living, *EQ-5D-3L* EuroQoL-5 dimension-3 level, *EQ-5D* EuroQoL-5 dimension, *FUP* follow-up, *ISs* Ischemic stroke survivors, *IQR* Inter quartile range, *NeuroQoL* Quality of Life in Neurological Disorders, *SS-QoL* Stroke Specific Quality of Life scale, *VAS* Visual analogue scale

The studies included in the review used different age limits to define young patients. The mean age of the participants ranged from 47 years [57] to 57 years [58] with the age range between 18 and 65 years. The upper limit of 50 years was used in two studies [20, 57], and the upper limit of 65 years was used in six studies [26, 54–56, 58, 60]. The upper limit of 55 years was used in one study [59].

### HRQoL between young adults with IS and age-matched controls

Differences in HRQoL between young adults with IS and age-matched controls from the general population were reported in four studies, in which generic measures (WHOQOL-BREF 26, EQ-5D) were applied. Young stroke patients rated significantly lower global health [59], physical functioning [58] and all domains of the EQ-5D, except pain/discomfort (Table 1).

### Instruments used for assessing the HRQoL

Seven outcome measures (Tables 1, 2, 4) were applied in the nine quantitative studies; five were generic (EQ-5D; EQ-5D-3L; WHOQOL-BREF 26) and four stroke-specific QoL measures (SS-QoL; SS-QoL-12; SIS; NeuroQoL). The most used measures were the EuroQoL instruments, which had been applied in four studies. Stroke-specific QoL measures had been applied in four studies [20, 55, 56, 60].

### Predictors of the HRQoL in young adults

Of all analysed studies, 14 variables (Table 3) potentially associated with the HRQoL were identified, and in most studies, several factors were examined in parallel. In most studies, multiple regression analyses were used (Table 3). Clinically related physical factors were more frequently addressed, followed by those centred on the psychosocial stroke sequels and the individual or sociodemographic factors. The severity of the stroke, functional status, or disability, independence in ADL, motor dysfunction, hand function, fatigue, and coronary heart disease during the index event were clinically related factors. Cognitive status, depression/anxiety, fear of stroke recurrence, return to work/unemployment, restrictions and limitations in leisure activities, self-perceived health status, and sense of coherence were psychosocial factors.

**Table 3:** Predictors of HRQoL in young adults with IS

Category	Factors	Measure	Statistical analyses	Main findings
Clinically related factors	Neurological outcomes, the severity of stroke	NIHSS	(SEM)	The severity of the stroke at the time of follow-up had significant direct, indirect and total effects on HRQoL [20]
	Functional outcomes, independence in ADL	mRS, BI	Regression model	Favourable initial functional status and higher independence in ADL: higher HRQOL [56]
			SEM	The functional status and higher independence in ADL at the time of follow-up had significant direct, and total effects on HRQoL [20]
	Motor impairment	MRC	Regression model	Lower motor dysfunction: higher HRQOL [56]
		SIS		
	Hand function	Upper extremity function of the SIS	Regression model	HRQoL at 12 months after stroke was predicted by hand function explaining a total of 32% of variance [56]
	Fatigue	FSS, FAS	Regression model	Higher levels of fatigue: lower HRQOL [57]
	Coronary heart disease during the index event		Regression model	No coronary heart disease during the index event: higher HRQOL [59]
Psychosocial factors	Depression	BDI, HADS, MADRS	Regression model	Higher levels of depression: lower HRQOL [57, 59]
			SEM	Depression at the time of follow-up had significant indirect, and total effects on HRQoL [20]
	Anxiety	HADS	Regression model	Higher levels of anxiety: lower HRQOL [57]
	Fear of stroke recurrence	FSRS	SEM	Fear of stroke recurrence at the time of follow-up had significant direct, indirect, and total effects on HRQoL [20]
	Restrictions and limitations in leisure activities	MYS questionnaire	Regression model	Higher levels of restrictions and limitations in leisure activities: lower HRQOL [58]
	Self-perceived health status	HPQ	SEM	General health perception had a significant direct effect on the HRQoL [20]
	Sense of coherence	SCS	Regression model	The higher initial sense of coherence: higher HRQOL [56]
	Return to work/unemployment		Regression model	Return to work: higher HRQoL
				Unemployment: lower HRQoL [20, 57–59]
	Social support	ENRICHD SSI	SEM	Higher social support: higher HRQOL
				Social support had significant direct, and total effects on HRQoL [20]

*BI* Barthel Index, *BDI* Beck Depression Inventory, *ENRICHD SSI* Coronary Heart Disease Patients Social Support Inventory, *FAS* Fatigue Assessment Scale, *FSS* Fatigue Severity Scale,– *FSRS* Fear of Stroke Recurrence Scale, *HPQ* Health Perception Questionnaire, *HADS* Hospital Anxiety and Depression Scale, *ADL* Activities of Daily Living, *MRC* Medical Research Council motor scale, *MMSE* Mini-Mental State Examination, *MMMS* Modified Mini-Mental State Examination, *mRS* Modified Rankin Scale, *MADRS* Montgomery-Åsberg Depression Rating Scale, *NIHSS* National Institutes of Health Stroke Scale, *SCS* Sense of Coherence scale, *SIS* Stroke Impact scale, *SEM* Structural equation modelling

Consistent results were reported for functional outcomes, independence in ADL, fatigue, depression, and return to work were demographic factors (Table 3). Poor functional outcomes, dysarthria, motor impairment and impaired hand function, depression/anxiety, fatigue, fear of stroke recurrence, coronary heart disease during the index event, and restrictions and limitations in leisure activities had a negative impact on the HRQoL in young adults with IS. Independence in ADL, social support, return to work, and higher general health perception had a positive influence on the HRQoL.

Three studies [55, 56, 60] confirmed a predictive value of clinical outcomes assessed using the modified Rankin Scale (mRS) and the Barthel index (BI) for the HRQoL. HRQoL had no significant association with mRS [57] in a long-term FUP. The impact of mRS on FUP was explained mostly by its association with physical functioning. However, in the study from the Netherlands, mRS in FUP did not significantly contribute to any domains of the HRQoL [57]. The proportion of independence in ADL based on BI ranged from 78% [60] to 84% [56] after a mean FUP of 6 to 12 months. Inconclusive results were found in the stroke severity. Stroke severity (scored by the National Institutes of Health Stroke Scale, NIHSS) was often reported at baseline (during the hospital admission or discharge) and two studies only provided the neurological deficits within months after discharge [20, 55]. The severity of persisting neurological deficit over time had negative effects on the HRQoL in the Korean study [20]. In the US study, the median of NIHSS score was significantly lower in the midlife stroke survivors’ group (45–64 years, median NIHSS: 2) than in the older group (≥ 65 years, median NIHSS: 3) [49]. The admission NIHSS had no significant impact on the HRQoL in stroke patients in the Netherlands study [57]. Three of the included studies assessed depression and/or anxiety [20, 57, 59], however, only in one of them, fatigue and depression were measured simultaneously (Table 3) [57].

## Discussion

The aim of this review was to investigate and map conceptually the current evidence in HRQoL in young adults. In comparison with the previous review [41], this scoping review contributes to the existing evidence with a deeper insight into what is known about the differences in the HRQoL poststroke between younger and older patients and about the relationship between functional outcomes and HRQoL in young adults.

Studies comparing the HRQoL between the younger and older stroke patients (Table 4) have produced inconsistent findings; the methods of the assessments of variables varied among studies and resulted in inconclusive data. Aspects of the HRQoL after stroke differed between younger and older individuals, most significantly in the functional outcomes and in the self-care [55, 56]. No significant difference was found in global recovery or global HRQoL [55, 56] and in the emotional aspects of the HRQoL [54, 56]. Between younger and older stroke survivors. Based on the available literature, it appears that the time of FUP and the type of HRQoL assessment may explain the inconsistent results regarding the differences in the HRQoL between younger and older individuals. Studies examining differences in the disease specific HRQoL between young and old patients provide consistent evidence that overall HRQoL assessed more than 12 months after IS was significantly higher in the younger patients (< 65 years) compared to older those [54, 56]. It was also well documented that young adults reported a higher level of self-care, mobility, functional outcome, and strength/energy 12 months after IS than old ones. However, inconsistent findings were found within the time of FUP ≤ 12 months [54, 55].

**Table 4:** Differences in QoL between younger and older individuals

Author/Year/Country	Study design	Participants and sampling	Age mean (SD), range or median (years)	HRQoL measure	Measure time (Time of assessment after stroke)	Main findings and conclusion
Lannin et al. (2017) [54] Australia	Retrospective study	Working age adults (18–64 years): 5154 ISs: 79% Older group (≥ 65 years):15,317	Not reported	EQ-5D-3L	90 and 180 days after hospital admission	The younger group reported problems with self-care (only 21%), performing usual activities (53%), and pain/discomfort (45%) less often compared with the older one. There were no statistical differences in mobility and emotional domain
Lisabeth et al. (2018) [55] USA	Retrospective study	The midlife ISs (45–64): 1618 Older ISs (≥ 65 years): 3240	The midlife ISs: 56.8 Median: 52.2 Older ISs: 78.3, Median: 72.4	SS-QoL-12	90 Days after IS	Despite more favourable 90-day neurologic, functional, and cognitive outcomes, the midlife stroke survivors’ group did not report better QoL than the old one
Palmcrantz et al. (2012) [56] Sweden	Prospective study	Young adult (< 65 years):63 (84% ISs) Older group (≥ 65 years):129	Young: 53 (11), 25–64 Old: 78 (8), 65–94	SIS	12 Months after IS	No statistical differences in global recovery between the groups. Young adults reported greater use of care and rehabilitation, higher levels of strength, self-care/domestic life, and mobility. Self-perceived global recovery in the younger group was mainly affected by hand function and depression. The initial sense of coherence, stroke severity and follow-up independence in ADL were associated with self-perceived global recovery in young adults at 12 months

*ADL* activities of daily living, *EQ-5D-3L* EuroQoL-5 dimension-3 level, *EQ-5D* EuroQoL-5 dimension, *ISs* Ischemic stroke survivors, *IQR* Inter quartile range, *mRS* Modified Rankin Scale, *SS-QoL-12* short-form Stroke Specific Quality of Life scale, *SIS* Stroke Impact scale, *VAS* Visual analogue scale

Aspects of HRQoL in the long-term FUP differ most significantly among young adults and their age-matched controls in physical functioning and in almost all domains of the EQ-5D. However, the studies comparing HRQoL between young stroke patients and their non-stroke counterparts (Table 2) predominantly addressed the perceived global health measured by generic instruments (the EQ-5D) and not QoL per se.

### Instruments used for assessing the HRQoL

However, only one instrument (the MYS questionnaire) was developed specifically to assess relevant aspects of the functioning and disability of young individuals with IS [58]. Conceptual and psychometric limitations of this instrument are the most important, mainly the lack of explicit, a priori models of the construct. For the measurement of post-stroke HRQoL, generic instruments were predominantly used. However, these measures do not comprehensively cover all relevant domains to HRQoL in young strokes. Therefore, there is a continuing need for examining the feasibility of methods to assess all domains of long-term consequences in patients with young stroke.

### Factors associated with the HRQoL in young adults

A wide spectrum of functional, and psychosocial factors related to the HRQoL has been investigated in many studies. In line with previous reviews [35, 61], various predictors of the HRQoL were identified in young adults with IS in this scoping review.

The incidence of poor functional outcomes in young adults at the time of hospital discharge is generally low [62]. Favourable functional status and low severity of the stroke were found to be the significant factors contributing to better long-term outcomes [21, 27, 63] and return to work [20, 63]. Several prospective and retrospective cohort studies on the long-term prognosis of young adults after IS have revealed that a substantial number of patients achieved independence in activities of daily living [21, 27, 63–65] and young patients achieved better clinical outcomes after IS in comparison with old ones [55, 56]. There are only a few studies that examined the associations between long-term functional outcomes and the HRQoL. Stroke severity (measured by the NIHSS) was not consistently associated with the HRQoL, but the NIHSS score was confirmed as the independent predictor of the HRQoL in several prospective studies. The predictability of the NIHSS score for the HRQoL was also observed in previous longitudinal studies measuring the HRQoL in the older populations at three [66], 12 months [66] or 2 years after IS [62]. Although NIHSS was identified as a predictor of good clinical outcomes in young, no prospective study assessed the impact of the baseline NIHSS on the HRQoL after IS in them. Thus, further studies related to changes in the severity status and functional outcomes over time (years after discharge) or their influence on the HRQoL in young adults are needed. In addition, the size effect of different functional outcome measures on the HRQoL was not also investigated. To our knowledge, some prospective studies in stroke survivors found that mRS is a better predictor of the HRQoL in long-term FUP than other scales (e.g. BI) [49, 66–68].

Getting sufficient evidence about long-term functional outcomes and remaining disability is strongly needed in young stroke survivors because of their long-life expectancy. Younger adults live with disabilities for a longer time, and their HRQoL is strongly influenced not only by independence in ADL. Young adults require and expect to achieve a higher level of functioning or independence in more complex roles because of their parenting and challenging family or work responsibilities [49, 56, 59]. Return to work (RTW) or the ability to stay at work significantly contributed to better HRQoL [20]. Post-stroke unemployment was assessed in six of the included studies and mainly using self-reported data. The Swedish study used data from the Swedish Health Insurance Office [26, 27]. In the Dutch study, young adults had a higher risk of post-stroke unemployment eight years after IS than their peers did [68]. Several predictors of RTW after IS (e.g., functional outcomes, stroke severity, fatigue, depression) reported in recent studies were like predictors of HRQoL identified in this review [25–27, 68, 69]. We could suggest a reciprocal relationship between the HRQoL and RTW after IS, which means that better HRQoL facilitates RTW and vice versa [25].

Psychological characteristics, namely post-stroke fatigue (PSF), depression (PSD) and anxiety (PSA) have proved the highest relevance as independent factors contributing to low HRQoL at a young age. Post-stroke depressive symptoms together with PSF have been recognised as common and persistent complaints jeopardizing the HRQoL after stroke [70–72]. Moreover, PSF [57] and PSD [57] among young adults correlated with the domains of the HRQoL stronger than functional outcomes (mRS). However, all the included studies used self-reported measures for assessing the level of fatigue, depression, and anxiety. The findings of this review emphasize the need to monitor PSF, PSD, and PSA also many years after IS and to improve awareness among healthcare professionals about their prevalence and impact on functional outcomes, the HRQoL or on return to pre-stroke activities among young stroke patients. The influence of PSD and PSF was observed even after a mean FUP of 5 to 6 years after IS [57]. Furthermore, PSD, PSA, and PSF had a negative influence on functional outcomes in young adults a decade after IS [16, 22]. Nevertheless, temporal relationships between PSD and PSF have not yet been reported. Pre-stroke depression significantly contributed to PSD [73].

In addition, the relationship between cognitive dysfunction or post-stroke pain and the HRQoL was not investigated in any study included in this review. However, the prevalence and course of cognitive dysfunction [6, 17–19] or post-stoke pain [74] in young IS patients have been examined in several studies.

The HRQoL was not measured repeatedly over time in most studies and only one pilot study compared the HRQoL in young patients between two-time points (baseline and 6 months after discharge) [60]. A significant improvement was found only in the independence in ADL and cognitive function, whereas the HRQoL did not improve over time [60]. Other studies examined the HRQoL only in FUP assessments (Table 1, 2, 3). Future research investigating the trajectories of the HRQoL domains and post-stroke symptoms at a young age is needed. In the Dutch quantitative study of 351 old IS patients (mean age of 67 ± 13 years), the trajectories of the HRQoL were investigated in the first 12 months after stroke [75]. The authors identified a four-trajectory model (high, low, recovery, and decline) for physical and psychosocial HRQoL and predictors of these diverse trajectories. Psychological factors (personality, coping competencies, illness cognitions, and self-efficacy) were the most significant factors in identifying individuals at risk of unfavourable post-stroke HRQoL outcomes [75]. In this review, we identified the following three psychosocial factors affecting the HRQoL: the sense of coherence [56], fear of stroke recurrence [20] and perceived social support [20]. Although the role and importance of psychological factors (such as personality, coping, locus of control, illness cognitions, self-efficacy, or self-worth) for the post-stroke HRQoL have been systematically studied, these factors have not been specifically examined in young stroke patients [29]. Indeed, psychological factors may influence the psychosocial adaptation post-stroke and therefore the perception of the HRQoL. The most examined predictors of post-stroke HRQoL in young adults were stroke-related factors (functional outcomes and PSD), and RTW. Further longitudinal studies are needed to identify the trajectory of post-stroke psychosocial symptoms over time and other potential predictors of unfavourable long-term HRQoL so that specific young stroke rehabilitation and stroke self-management support programmes could be developed.

Most of the studies included in this review were from Western countries, and only two studies were conducted outside of this area (South Korea). A paucity of studies conducted in other parts of World (especially in developing countries) remains, however, epidemiological evidence published in the recent narrative reviews [1, 5] has highlighted that data on incidence and prevalence are still scarce for many African and Asian countries.

### Study limitations

Cohen’s kappa was not used to calculate concurrence between authors. Disagreement was solved only by the joint discussion until a consensus was reached. The search was limited to the electronic scientific databases accessible to the authors’ institution. The comparative analysis of included studies was limited, mainly due to the different study designs across studies and inherent heterogeneity in the subtypes of stroke. For measuring the HRQoL, different tools were used, mainly generic instruments. In addition, the HRQoL was measured at a different time of FUP, and a varying number of variables were used in the included studies. These factors could contribute to inconclusive findings. This scoping review suggested that the HRQoL in young adults with IS can be affected mainly by commonly used stroke clinical outcomes (measured by mRS and BI) and psychological factors (post-stroke fatigue and depression).

## Conclusion

There is still a gap in the evidence for the HRQoL outcomes after IS and in the role of psychosocial variables for the HRQoL in young IS patients. The reviewed studies emphasized the importance of functional outcomes, post-stroke depression, fatigue and anxiety and early return to work. The findings of our review can provide deeper insight and a better understanding of the various factors contributing to the long-term HRQoL after IS and may support the development of specific interventions for stroke self-management programmes. Further large prospective studies focusing on the factors affecting the HRQoL in young patients after IS are warranted.

## Supplementary Information

**Additional file 1.** Detailed search strategy in databases.

## Data Availability

Not applicable.
