# Supplementary material for: Factors affecting the quality of life after ischemic stroke in young adults: a scoping review
Source: Health Qual Life Outcomes. 2023 Jan 19;21:4. doi: 10.1186/s12955-023-02090-5 (PMC9850784; doi:10.1186/s12955-023-02090-5)
Supplement: Supplementary file 1 — Additional file 1. Detailed search strategy in databases. [file 12955_2023_2090_MOESM1_ESM.docx]

The table below shows concepts and terms searched both as controlled vocabulary (MeSH in MEDLINE, PsycINFO terms in PsycINFO etc.) and as keywords. Manual searching of the results will remove unnecessary or irrelevant records.

**Detailed example: MEDLINE and PsychInfo search**

|  | **Stroke** | **Quality of life** | **Young adult** |
| --- | --- | --- | --- |
| **Controlled vocabulary: MEDLINE MeSH** | exp Ischemic Stroke | exp Quality of Life | exp Young Adult |
| Keywords | (acute ischemic stroke or acute ischemic strokes or ischaemic stroke or ischaemic strokes or ischemic stroke or ischemic stroke, acute or ischemic strokes or stroke, acute ischemic or stroke, ischaemic or stroke, ischemic).mp | (hrqol or "health related quality of life" or "health-related quality of life" or life quality or "quality of life").mp | (adult, young or adults, young or young adult or young adults).mp. |
| **Controlled vocabulary: APA Thesaurus of Psychological Index Terms** | DE "Cerebrovascular Accidents" | DE "Quality of Life" AND DE "Health Related Quality of Life" |  |
| Keywords | stroke OR cerebrovascular stroke OR cerebrovascular accident OR ischemic stroke OR ischaemic stroke | quality of life or well-being or well-being or health-related quality of life | young OR adult, young OR young adult OR middle-aged adults OR working age OR stroke patient OR post-stroke OR stroke survivor |

| **DATABASE** | **Search strategy** |
| --- | --- |
| **Ovid MEDLINE**  Searched on May 11, 2021  Limit to 2000 to present and English language | #1  ((ischemic stroke OR (acute ischemic stroke.mp OR acute ischemic strokes.mp OR ischaemic stroke.mp OR ischaemic strokes.mp OR ischemic stroke.mp OR ischemic stroke, acute.mp OR ischemic strokes.mp OR stroke, ischaemic.mp OR stroke, ischemic.mp) AND (adult, young.mp OR adults, young.mp OR young adult.mp OR young adults.mp) AND ("Quality of Life"/ OR "health-related quality of life".mp) OR (hrqol.mp OR "health related quality of life".mp OR "health-related quality of life".mp OR life quality.mp OR "quality of life".mp))  **2 records retrieved**  #2  ((stroke or cerebrovascular stroke or cerebrovascular accident or ischemic stroke or ischaemic stroke) and (quality of life or health-related quality of life or health status or functional status or activities of daily living or functional disability) and (young or adult, young or young adult or middle-aged adults or working age or stroke patient or post-stroke or stroke survivor)).ab.  **1990 records retrieved**  #3  ((stroke or cerebrovascular stroke or cerebrovascular accident or ischemic stroke or ischaemic stroke) and (quality of life or health-related quality of life or health status or functional status or activities of daily living or functional disability) and (young or adult, young or young adult or middle-aged adults or working age or stroke patient or post-stroke or stroke survivor)).ti.  **147 records retrieved** |
|  |  |
| **EBSCO MEDLINE**  Searched on May 18, 2021  Limit to 2000 to present and English language | #1  AB ( stroke OR cerebrovascular stroke OR cerebrovascular accident OR ischemic stroke OR ischaemic stroke ) AND AB ( quality of life OR health-related quality of life OR health status OR functional status OR activities of daily living OR functional disability ) AND AB ( young OR adult, young OR young adult OR middle-aged adults OR working age OR stroke patient OR post-stroke OR stroke survivor )  **9211 records retrieved**  #2  TI ( stroke OR cerebrovascular stroke OR cerebrovascular accident OR ischemic stroke OR ischaemic stroke ) AND TI ( quality of life or well being or well-being or health-related quality of life ) AND TI ( young OR adult, young OR young adult OR middle-aged adults OR working age OR stroke patient OR post-stroke OR stroke survivor)  **443 records retrieved** |
| **PsycINFO via EBSCO**  Searched on May 18, 2021  Limit to 2000 to present and English language | #1  AB ( stroke OR cerebrovascular stroke OR cerebrovascular accident OR ischemic stroke OR ischaemic stroke ) AND AB ( quality of life or well-being or well-being or health-related quality of life ) AND AB ( young OR adult, young OR young adult OR middle-aged adults OR working age OR stroke patient OR post-stroke OR stroke survivor )  **668records retrieved**  #2  TI ( stroke OR cerebrovascular stroke OR cerebrovascular accident OR ischemic stroke OR ischaemic stroke ) AND TI ( quality of life or well being or well-being or health-related quality of life ) AND TI ( young OR adult, young OR young adult OR middle-aged adults OR working age OR stroke patient OR post-stroke OR stroke survivor)  **324 records retrieved** |
| **ProQuest Science Database**  Searched on June 25, 2021  Limit to 2000 to present and English language | #1  mainsubject.Exact("health-related quality of life" OR "quality of life") AND mainsubject.Exact("ischemic stroke patients" OR "ischemic strokes" OR "acute ischemic stroke" OR "health-related quality of life" OR "quality of life" OR "ischemic stroke") AND mainsubject.Exact "young adults"  #2  **269 records retrieved**  ab(cerebrovascular accident or brain attack or stroke) AND ab(quality of life OR health-related quality of life OR health status OR functional status OR activities of daily living OR functional disability) AND ab(young stroke patients)  **269 records retrieved** |

**Detailed example: MEDLINE search**

1.(acute ischemic stroke or acute ischemic strokes or ischaemic stroke or ischaemic strokes or ischemic stroke or ischemic stroke, acute or ischemic strokes or stroke, acute ischemic or stroke, ischaemic or stroke, ischemic).mp. [mp=title, abstract, original title, name of substance word, subject heading word, floating sub-heading word, keyword heading word, organism supplementary concept word, protocol supplementary concept word, rare disease supplementary concept word, unique identifier, synonyms]

limit to yr="2000 -Current"

2.(hrqol or "health related quality of life" or "health-related quality of life" or life quality or "quality of life").mp. [mp=title, abstract, original title, name of substance word, subject heading word, floating sub-heading word, keyword heading word, organism supplementary concept word, protocol supplementary concept word, rare disease supplementary concept word, unique identifier, synonyms]

limit 15 to yr="2000 -Current"

3.(adult, young or adults, young or young adult or young adults).mp. [mp=title, abstract, original title, name of substance word, subject heading word, floating sub-heading word, keyword heading word, organism supplementary concept word, protocol supplementary concept word, rare disease supplementary concept word, unique identifier, synonyms]

limit 17 to yr="2001 -Current"

1 and 2 and 3
